# Supplementary material for: Virtual Screening, Structural Analysis, and Formation Thermodynamics of Carbamazepine Cocrystals
Source: Pharmaceutics. 2023 Mar 3;15(3):836. doi: 10.3390/pharmaceutics15030836 (PMC10052035; doi:10.3390/pharmaceutics15030836)
Supplement: Supplementary file 1 [file pharmaceutics-15-00836-s001.zip › pharmaceutics-2251144-supplementary.pdf]

Supplementary Materials: Virtual screening, structural analysis and formation thermodynamics of carbamazepine cocrystals

Artem O. Surov , Anna G. Ramazanova, Alexander P. Voronin, Ksenia V. Drozd, Andrei V. Churakov and German L. Perlovich

Table S1. List of the coformers used for screening and results of the experimental cocrystal screening.

CID	Molecular structure	Name	Outcome
464	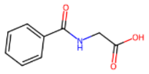	Hippuric acid	Physical mixture
1047	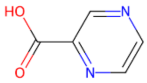	Pyrazine-2-carboxylic acid	Physical mixture
3282	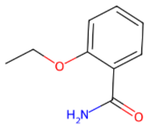	Ethenzamide	Physical mixture
4386	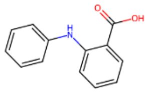	N-phenylanthranilic acid	Physical mixture
**6971**	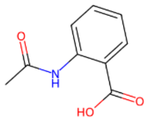	**2-Acetamidobenzoic acid**	**Cocrystal**
7470	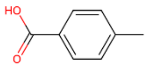	4-Methylbenzoic acid	Physical mixture
7478	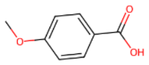	4-Methoxybenzoic acid	Physical mixture
11370	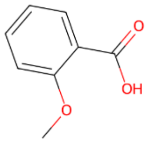	2-Methoxybenzoic acid	Physical mixture
14533	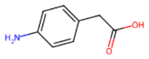	4-Aminophenylacetic acid	Physical mixture

**19266**	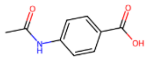	**4-Acetamidobenzoic acid**	**Cocrystal**
**48847**	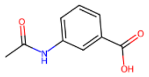	**3-Acetamidobenzoic acid**	**Cocrystal**
66345	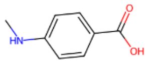	4-(Methylamino)benzoic acid	Physical mixture
76079	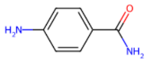	4-Aminobenzamide	Physical mixture
108776	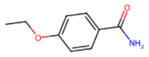	4-Ethoxybenzamide	Physical mixture
445858	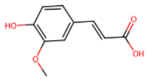	Ferulic acid	Physical mixture
637775	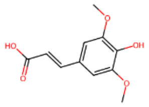	Sinapic acid	Physical mixture
689043	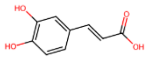	Caffeic Acid	Physical mixture

Table S2. List of calculated score values for different virtual screening models for experimentally tested CBZ – coformer pairs (87 cases).

CID	Coformer	CCGNet	HBP	MEP method A	MEP method B	experimental outcome	Refcode or ref.
19	2,3-Dihydroxybenzoic acid	37.2	-0.09	2	10	yes	LEXQAJ
135	4-Hydroxybenzoic acid	-22.7	-0.09	5.9	11.7	yes	MOXVIF
178	Acetamide	34.7	0.01	-2.3	4	yes	[1]
196	Adipic Acid	-32	0.06	1.8	6.2	yes	MOXVEB
227	Anthranilic acid	42.8	0.09	6.5	8.4	yes	RUTGOE
243	Benzoic Acid	41.6	-0.05	3.6	5.4	yes	MOXVAX
338	Salicylic Acid	41.3	-0.1	10.8	11.1	yes	MOXWAY
385	Pimelic acid	-30.1	0.06	1.8	6.2	no	[1]
464	Hippuric acid	42.7	-0.1	4.6	4.7	no	This work
525	Malic acid	-33.7	-0.05	-	-	yes	[2]
723	2-Butenedioic acid	43.1	-0.02	4.7	9.5	yes	WEYFEN
743	Glutaric acid	-14.2	0.05	3.2	7.7	yes	MOXVOL
757	Glycolic acid	-8.3	-0.02	8.4	9.9	no	[2]
785	Hydroquinone	43.9	-0.15	4.6	9.9	yes	ABOQUF
867	Malonic acid	7.5	0.06	4.2	9.8	yes	MOXVUR
875	Tartaric acid	-16.5	-0.07	2.9	12.7	yes	MOXWIG01
932	5,7-Dihydroxy-2-(4-hydroxyphenyl)chroman-4-one	38.5	-0.2	4.7	10.9	yes	TOJSOD
936	Nicotinamide	46.1	0.03	-1.8	3.5	yes	UNEZES

938	Nicotinic acid	-19.9	-0.04	7.2	8.3	no	[3]
971	Oxalic Acid	37.7	-0.11	3.5	12.1	yes	MOXWUS
978	4-Aminobenzoic acid	43.8	0.1	2.5	8.2	yes	XAQRAJ
1046	Pyrazinamide	-12.9	0.03	0.8	2.2	no	[4]
1047	Pyrazine-2-carboxylic acid	15.3	0.04	-0.4	4.2	no	This work
1110	Succinic Acid	-18.2	0.05	1.5	6.9	yes	XOBCIB
1491	2,4-Dihydroxybenzoic acid	45.5	-0.09	5.6	12.6	yes	JANZUX
1983	Acetaminophen	-7.5	-0.01	0.5	7.9	no	[5]
2153	Theophylline	-8.8	-0.08	5.4	8.1	no	[5]
2201	9-Anthracenecarboxylic acid	41.7	-0.13	5.9	6.4	yes	LEXPIQ
2244	Aspirin	28.6	-0.05	6	6.4	yes	TAZRAO
2266	Azelaic acid	-24.1	0.08	1.1	5.5	no	[1]
2331	Benzamide	-18	0.07	-0.3	3.2	yes	WEKDID
2519	Caffeine	-17.9	0.1	2.1	4.1	no	[5]
3220	Emodin	41.5	-0.02	11.7	12	yes	[6]
3282	Ethenzamide	38.1	0.1	-0.2	0.9	no	This work
3469	2,5-Dihydroxybenzoic acid	39	-0.09	6.9	11.6	yes	JAPBAH
3715	Indomethacin	14.1	-0.06	7	8.9	yes	LEZKEI
3767	Isoniazid	44.1	0.19	1.9	4.3	no	[1]
3825	Ketoprofen	13.6	-0.11	8.7	9	yes	RAFGEO
4386	N-phenylanthranilic acid	37.8	-0.13	7	7.5	no	This work
4485	Nifedipine	44.5	-0.03	8.8	9	yes	WEKDUP
4649	4-Aminosalicylic acid	38.3	0.08	4.7	10	yes	FAYXOV
4650	1,4-Benzoquinone	46.3	-0.06	-3.9	2.2	yes	UNEYOB
5143	Saccharin	41.8	0.04	8	9.6	yes	UNEZAO
5147	Salicylamide	-28.2	0.07	2.5	6.4	no	[4]
5192	Sebacic acid	-22	0.08	1.1	5.5	no	[1]
6409	2,2,2-Trifluoroethanol	42.5	0.12	5.1	7.7	yes	SAPDUJ
6844	1-Hydroxy-2-naphthoic acid	35.6	-0.11	11	11	yes	MOXWEC
6971	N-Acetylanthranilic acid	30.9	-0.12	5.2	5.3	yes	This work
7064	6-Phenyl-1,3,5-triazine-2,4-diamine	45.1	0.15	-0.8	7.7	yes	BIXBOC
7433	3,5-Dinitrobenzoic acid	-22.6	-0.17	13.7	18.7	yes	HORWIX
7456	Methylparaben	29.5	-0.14	6.3	7.2	yes	HEDYAU
7470	4-Methylbenzoic acid	21.4	-0.05	3.4	4.5	no	This work
7478	4-Methoxybenzoic acid	6.8	-0.01	3.4	3.8	no	This work
7978	2-Aminopyrimidine	-2	0.11	-1.5	1.1	yes	JIQLAZ
8456	2-tert-Butyl-4-methoxyphenol	42.3	-0.1	8.8	9.2	yes	HEDYIC
8567	Meglumine	-37.5	0.1	-6.5	8.2	no	[7]
9338	2,6-Dihydroxybenzoic acid	26.6	-0.1	13.4	14.1	yes	JANZOR
10367	2,6-Pyridinedicarboxylic acid	27.6	-0.11	0.5	9.6	yes	XAQRIR
10457	Suberic acid	-40.2	0.07	1.5	5.7	no	[1]
11092	Paeonol	39.3	-0.09	-2	3.2	yes	[6]
11107	4,4'-Bipyridine	5.9	0.16	0.2	4	yes	XAQQUC
11112	Nicotinic acid hydrazide	44.8	0.18	1.7	4.6	no	[1]
11370	2-Methoxybenzoic acid	9.6	-0.01	0.7	1	no	This work
14300	4-Nitropyridine N-oxide	45.5	0.17	-2.9	3.1	yes	JIQKUS
14533	4-Aminophenylacetic acid	32.2	0.21	1.9	5.2	no	This work
15070	Picolinamide	-49.1	0.04	-0.7	0.6	no	[8]
15074	Isonicotinamide	-23.2	0.03	-0.4	4.2	yes	LOFKIB
19266	4-Acetamidobenzoic acid	24.8	-0.11	0.1	5.4	yes	This work
21954	Maleic hydrazide	-2.1	-0.01	1.7	7.1	no	[1]
48847	3-Acetamidobenzoic acid	23.3	-0.12	-0.9	5.8	yes	This work
65052	4-Hydroxybenzamide	45.8	0.11	0.6	7.1	yes	SOGSEP
66345	4-(Methylamino)benzoic acid	39.8	-0.01	3	6.7	no	This work
69021	2-Hydroxyacetamide	5.6	0.09	-0.6	5.5	no	[7]
72073	Quindoxin	42.3	0.16	1.4	3.2	yes	VIGGOI
76079	4-Aminobenzamide	-31.3	0.12	-1.6	5.6	no	This work
94220	Lactamide	4.3	0.06	-0.4	3.3	no	[7]
108776	4-Ethoxybenzamide	36.8	0.08	0.5	1.1	no	This work
222656	DL-malic acid	-37.2	-0.06	4.4	11.1	yes	[2]
444266	Maleic acid	6.6	-0.02	6.7	7.8	yes	MOXWOM
444539	Cinnamic acid	39.3	-0.04	3.5	4.3	yes	[9]
445858	Ferulic acid	21.3	-0.12	1.3	6.3	no	This work
520103	Pyrazine, 1,4-dioxide	6.3	0.2	-6	3.2	yes	VIGGUO
637775	Sinapic acid	41.3	-0.09	-0.3	3.5	no	This work
643757	cis-Aconitic acid	15.4	0.04	-2.2	10	yes	[3]
689043	Caffeic Acid	42.3	-0.1	-1.4	12.5	no	This work
2723790	Thiourea	46.9	0.02	2.3	11.7	yes	UWAZID
3035171	Benzo[d]isothiazole-3(2H)-thione 1,1-dioxide	43.1	-0.02	9.1	9.8	yes	YAJGEY
5281727	Pterostilbene	40.3	-0.12	9.5	13.5	yes	YABHIU

Table S3. Experimental data and crystallographic information for [CBZ+3AcAmBA] (1:1), [CBZ+4AcAmBA] (1:1) and 2AcAmBA ethyl acetate solvate.

Compound	[CBZ+3AcAmBA] (1:1)	[CBZ+4AcAmBA] (1:1)	2AcAmBA ethyl acetate solvate
Crystal data			
Chemical formula	$C_{15}H_{12}N_2O{\cdot}C_9H_9NO_3$	$C_{15}H_{12}N_2O{\cdot}C_9H_9NO_3$	$C_9H_9NO_3$
M_r	415.44	415.44	179.17
Crystal system, space group	Monoclinic, *P*2$_1$/*n*	Monoclinic, *P*2$_1$/*c*	Monoclinic, *C*2/*c*
Temperature (K)	100	150	150
a, *b*, *c* (Å)	7.0272 (3), 11.4177 (4), 25.3995 (10)	6.7920 (2), 24.9978 (8), 12.1120 (4)	14.3522 (12), 18.7197 (16), 7.4273 (6)
β (°)	97.7026 (15)	94.9744 (12)	
V (Å^3)	2019.53 (14)	2048.69 (11)	92.155 (3)
Z	4	4	8
Radiation type		Mo Kα	
μ (mm−1)	0.10	0.09	0.09
Crystal size (mm)	0.25 × 0.15 × 0.10	0.25 × 0.25 × 0.20	0.15 × 0.15 × 0.01
	Data collection		
Diffractometer	Bruker D8 Venture	Bruker D8 Venture	Bruker D8 Venture
Absorption correction	Multi-scan *SADABS* (Bruker, 2016)	Multi-scan *SADABS* (Bruker, 2016)	Multi-scan *SADABS* (Bruker, 2016)
T_{min}, T_{max}	0.871, 0.928	0.883, 0.928	0.837, 0.928
No. of measured, independent and observed [I > 2σ(I)] reflections	26443, 4407, 3385	29190, 4922, 4316	11010, 1856, 1327
R_{int}	0.055	0.029	0.047
(sin θ/λ)max (Å−1)	0.639	0.661	0.606
	Refinement		
R[F2 > 2σ(F2)], wR(F2), S	0.041, 0.091, 1.02	0.039, 0.102, 1.04	0.041, 0.117, 1.02
No. of reflections	4407	4922	1856
No. of parameters	365	365	154
H-atom treatment	All H-atom parameters refined	All H-atom parameters refined	All H-atom parameters refined
$\Delta\rho_{max}$, $\Delta\rho_{min}$ (e Å^{-3})	0.23, -0.18	0.35, -0.17	0.43, −0.19

Table S4. Metric and energetic parameters for conventional hydrogen bonds in [CBZ+3AcAmBA] (1:1) and [CBZ+4AcAmBA] (1:1).

Crystal	[CBZ+3AcAmBA] (1:1)				[CBZ+4AcAmBA] (1:1)			
Fragment	D(*X*···*A*)	D(H···*A*)	∠(*D*—H···*A*)	E_{int}	D(*X*···*A*)	D(H···*A*)	∠(*D*—H···*A*)	E_{int}
O1—H1···O3^{i}	2.6182 (16)	1.76 (2)	160 (2)	42.2	2.6046 (13)	1.70 (2)	161 (2)	45.4
N1—H1···O11	2.8430 (16)	1.950 (19)	169.3 (17)	28.5	2.8676 (13)	1.991 (17)	167.3 (15)	27.0
N11—H11···O11^{ii}	2.9248 (17)	1.991 (19)	178.2 (16)	24.2	2.9017 (13)	2.014 (17)	175.5 (15)	26.5
N11—H12···O2^{iii}	2.8768 (16)	2.088 (19)	146.7 (16)	20.9	2.9737 (14)	2.193 (16)	147.2 (14)	17.5

[a] Distances between the donor and acceptor atoms D(X···A) and between the hydrogen and acceptor atom D(H···A) in Å, where X =O, N and A = O, N, angle ∠(X-H···A) in º, the interaction energy (E_{int}) obtained from eq. (2) in kJ mol^{-1}.
Symmetry codes: *(i) x+1, -y+1/2, z-1/2; (ii) -x+1, -y+1, -z+2; (iii) -x+1, y+1/2, -z+3/2.*

Table S5. Experimental values of solubility product (K_{sp} in (mol·L^{-1})^2) for the selected [CBZ + CF] (1:1) cocrystals, intrinsic solubility of CBZ (S_0(CBZ) in mol·L^{-1}) and coformer (S_0(CF) in mol·L^{-1}) in different solvents and the calculated values of the Gibbs energy of the cocrystallization reaction ($\Delta_{form}G$ in kJ·mol^{-1}).

Coformer	Solvent	log(K_{sp})	log(S_0(CBZ))	log(S_0(CF))	$\Delta_{form}G$	Ref
Emodin	Ethanol	-4.28	-0.97	-1.74	-8.91	[6]
Emodin	2-propanol	-4.82	-1.36	-1.68	-10.21	[6]
Emodin	Ethyl acetate	-4.64	-1.32	-1.43	-10.77	[6]
Emodin	Acetone	-4.07	-1.27	-1.29	-8.58	[6]
Paeonol	2-propanol	-2.32	-1.36	-0.12	-4.83	[6]
Paeonol	Ethanol	-1.74	-0.97	0.08	-4.84	[6]
Paeonol	Methanol	-0.84	-0.49	0.22	-3.19	[6]
Nicotinamide	Ethyl acetate	-3.17	-1.31	-0.96	-5.17	[10]
Nicotinamide	Ethanol	-1.66	-0.85	0.04	-4.82	[10]
Nicotinamide	2-propanol	-2.43	-1.3	-0.2	-5.31	[10]
Glutaric acid	Ethyl acetate	-2.05	-1.31	0	-4.23	[10]
Glutaric acid	Ethanol	-1.03	-0.85	0.45	-3.54	[10]
Glutaric acid	2-propanol	-1.31	-1.3	0.56	-3.23	[10]

Table S6. Solubility values of carbamazepine and isomers of acetamidobenzoic acid in the selected organic solvents. The solubility ratio ($C_{p,CBZ}/C_{p,coformer}$) is shown in parenthesis.

Solvent	$C_{p,CBZ}\cdot10^2$	$C_{p,2AcAmBA}\cdot10^2$	$C_{p,3AcAmBA}\cdot10^2$	$C_{p,4AcAmBA}\cdot10^2$
Acetonitrile	14.2±0.3	2.50±0.08<br>(5.7)	0.43 ± 0.10<br>(33.0)	1.01±0.01<br>(14.1)
Methanol	22.0±0.2	-	7.50±0.05<br>(2.9)	-

Table S7. Solubility of CBZ, 4AcAmBA and cocrystal in acetonitrile at different temperatures, values of $\ln K_f$ and the Gibbs energy associated with the [CBZ+4AcAmBA] (1:1) cocrystal formation.

T	$C_{p,CBZ}\cdot10^2$	$C_{p,4AcAmBA}\cdot10^2$	$C_{cc,CBZ}\cdot10^2$	$C_{cc,4AcAmBA}\cdot10^2$	$\ln K_f$	$\Delta_{form}G$
K			mol/l			kJ·mol^{-1}
293.15	14.2±0.3	1.010±0.010	0.875±0.014	0.880±0.016	2.93±0.04	-7.13±0.09
298.15	16.2±0.4	1.145±0.005	1.013±0.015	1.046±0.010	2.86±0.03	-7.09±0.08
303.15	19.1±0.4	1.280±0.007	1.198±0.007	1.222±0.017	2.82±0.03	-7.10±0.07
308.15	22.2±0.3	1.473±0.007	1.428±0.009	1.472±0.008	2.75±0.02	-7.04±0.05
313.15	25.0±0.4	1.660±0.030	1.684±0.022	1.707±0.030	2.67±0.03	-6.95±0.08

Table S8. Solubility of CBZ, 3AcAmBA and cocrystal in methanol at different temperatures, values of $\ln K_f$ and the Gibbs energy associated with the [CBZ+3AcAmBA] (1:1) cocrystal formation.

T	$C_{p,CBZ}\cdot10^2$	$C_{p,3AcAmBA}\cdot10^2$	$C_{cc,CBZ}\cdot10^2$	$C_{cc,3AcAmBA}\cdot10^2$	$\ln K_f$	$\Delta_{form}G$
K		mol/l				kJ·mol^{-1}
293.15	22.0±0.2	7.50±0.05	7.00±0.06	7.0±0.1	1.20±0.02	-2.93±0.05
298.15	24.8±0.2	8.70±0.05	8.20±0.10	8.1±0.1	1.17±0.02	-2.91±0.05
303.15	28.5±0.2	9.70±0.05	9.50±0.08	9.5±0.1	1.12±0.02	-2.83±0.05
308.15	32.3±0.3	10.90±0.08	11.00±0.08	11.1±0.06	1.06±0.015	-2.72±0.04
313.15	35.6±0.1	11.95±0.06	12.10±0.08	12.5±0.07	1.04±0.02	-2.69±0.03

Table S9. Reported experimental thermodynamic parameters for cocrystallization reactions.

N	Cocrystal	T<br>K	$\Delta_{form}G$	$\Delta_{form}H$<br>kJ·mol^{-1}	$T\Delta_{form}S$	Ref.
1	[Bicalutamide + Benzamide] (1:1)	298	-3.4±0.3	-19.6±0.8	-16.2±1.1	[11]
2	[Bicalutamide + Salicylamide] (1:1)	298	-2.2±0.3	-11.4±0.5	-9.2±0.8	[11]
3	[**XI**[a]+Vanillic acid] (1:1)	298	-5.3±0.4	-10.5±0.9	-5.2±1.3	[12]
4	[Salinazid + Saccharin] (1:1)	298	-7.3±0.6	-18.4±1.0	-11.1±1.6	[13]
5	[Vanillin isoniazid + Saccharin] (1:1)	298	-9.1±0.8	-13.5±1.1	-4.4±1.9	[13]
6	[4-Aminobenzoic acid +Pyrazinamide] (1:1)	298	-6.1±0.3	-39.6±2.9	-33.5±3.2	[14]
7	[CBZ + Saccharin] (1:1)	306	-4.6	-5.9±0.9	-1.3	[15]
8	[Adefovir + Saccharin] (1:1)	293	-12.1	-41.6±6.0[b]	-29.5[b]	[16]
9	[Glycine + Fumaric acid] (2:1)	298	-4.4±0.3	-5.8±0.5	-1.4±0.6	[17]
10	[Glycine + Maleic acid] (1:1)	298	-5.4±0.4	-11.1±0.9	-5.7±1.0	[17]
11	[Sorafenib + 5-fluorouracil] (1:1)	303	-4.78±0.09	-4.76±0.07	0.02±0.1	[18]
12	[Nitrofurantoin + Picolinamide] (1:1)	298	-4.9 ± 0.5	-4.4 ± 0.7	0.6 ± 0.8	[19]
13	[Nitrofurantoin + 2-Aminobenzamide] (1:1)	298	-4.5 ± 0.5	-3.3 ± 0.8	1.3 ± 1.1	[19]
14	[Nitrofurantoin + Salicylamide] (1:1)	298	-0.6 ± 0.4	-0.3 ± 0.6	0.3 ± 0.7	[19]
15	[Nitrofurantoin + Isonicotinamide] (1:1)	298	-4.7 ± 0.4	1.3 ± 0.6	6.0 ± 0.7	[19]
16	[Celecoxib + Nicotinamide] (1:1)	298	-0.35±0.05	6.5±1.5	6.9±2.0	[20]
17	[Sulfamethazine + salicylic acid] (1:1)	293	-7.1	23.1	29.7	[21]
18	[CBZ + Benzamide] (1:1)	298	-4.8±0.3	25.7 ± 1.9	30.5 ± 2.9	[22]
19	[CBZ + 4-Hydroxybenzamide] (1:1)	298	-6.7 ± 0.5	-12.4 ± 0.9	-5.7 ± 0.5	[22]
20	[CBZ + isonicotinamide] (1:1)	298	-2.1 ± 0.1	19.9 ± 1.9	22.0 ± 2.5	[22]
21	[CBZ+3AcAmBA] (1:1)	298	-2.91±0.05	-6.7±0.5	-3.8±0.5	This work
22	[CBZ+4AcAmBA] (1:1)	298	-7.09±0.08	-9.5±0.6	-2.4±0.6	This work

[a]XI - 1-[5-(3-Chloro-phenylamino)-1,2,4-thiadiazol-3-yl]-propan-2-ol. [b] Calculated using data provided in the original paper.

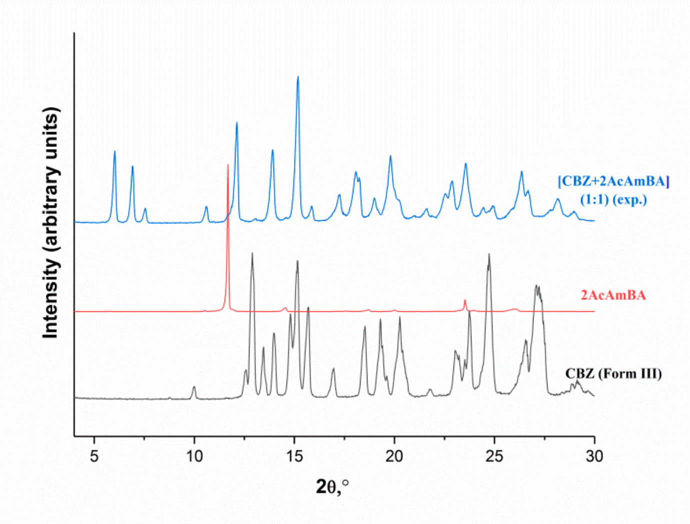

(**a**)

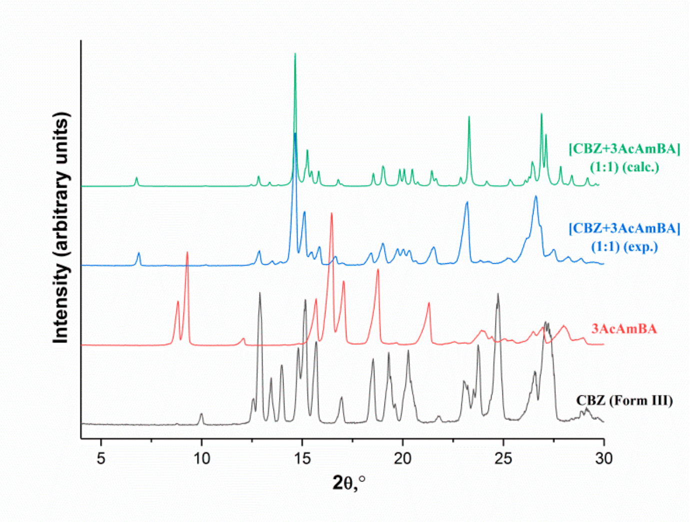

(**b**)

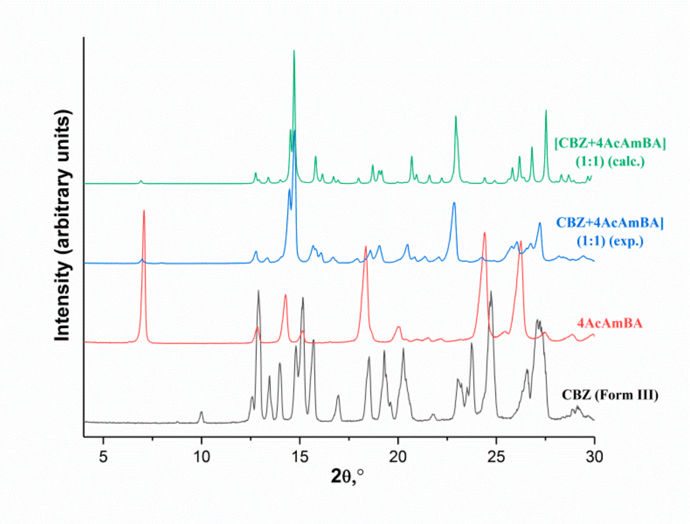

(**c**)

Figure S1. Experimental PXRD patterns of CBZ, 2AcAmBA, 3AcAmBA, 4AcAmBA and the cocrystals obtained via mechanochemical treatment: (a) [CBZ+2AcAmBA] (1:1), (b) [CBZ+3AcAmBA] (1:1), (c) [CBZ+4AcAmBA] (1:1).

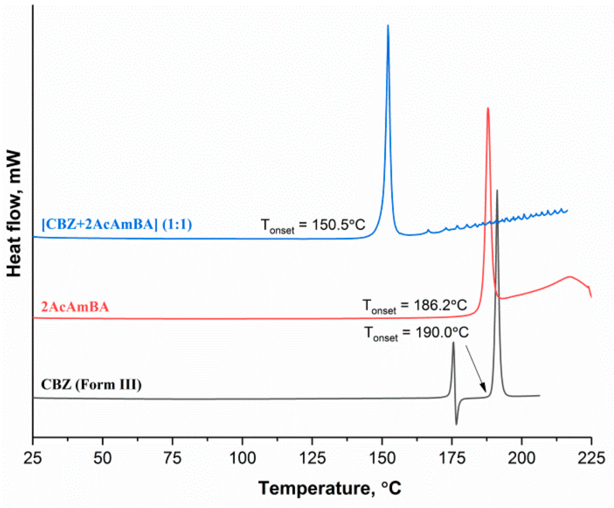

(**a**)

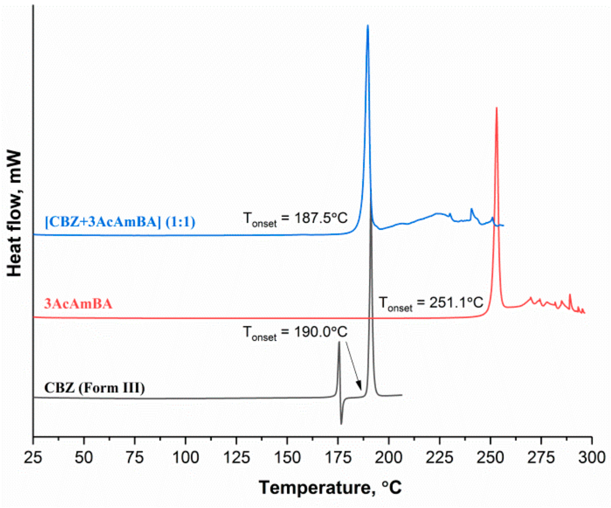

(**b**)

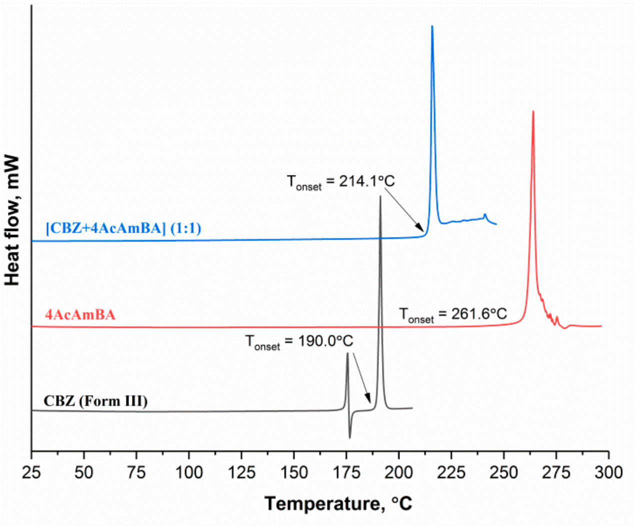

(**c**)

Figure S2. Experimental DSC curves for CBZ, 2AcAmBA, 3AcAmBA, 4AcAmBA and the obtained cocrystals: (a) [CBZ+2AcAmBA] (1:1), (b) [CBZ+3AcAmBA] (1:1), (c) [CBZ+4AcAmBA] (1:1).

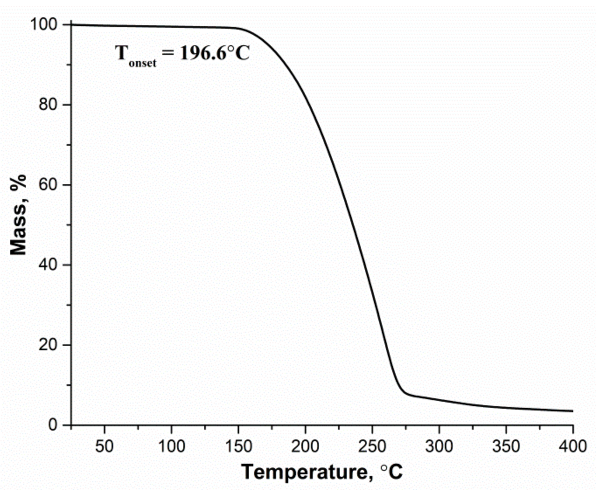

(**a**)

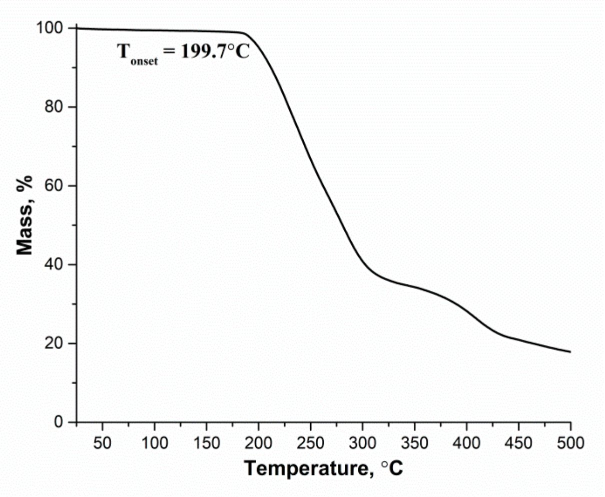

(**b**)

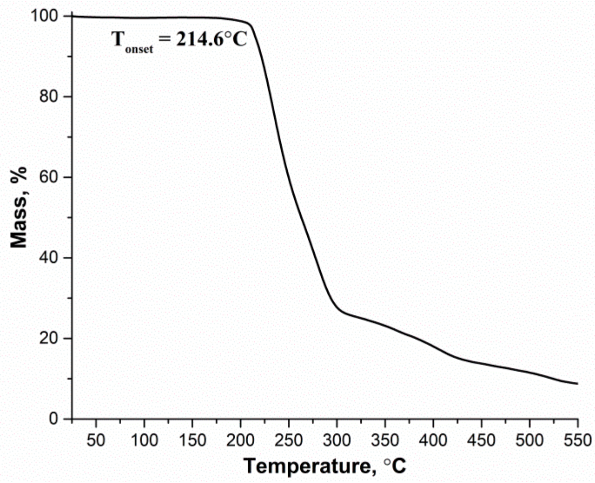

(**c**)

Figure S3. Experimental TGA curves for the obtained cocrystals: (a) [CBZ+2AcAmBA] (1:1), (b) [CBZ+3AcAmBA] (1:1), (c) [CBZ+4AcAmBA] (1:1).

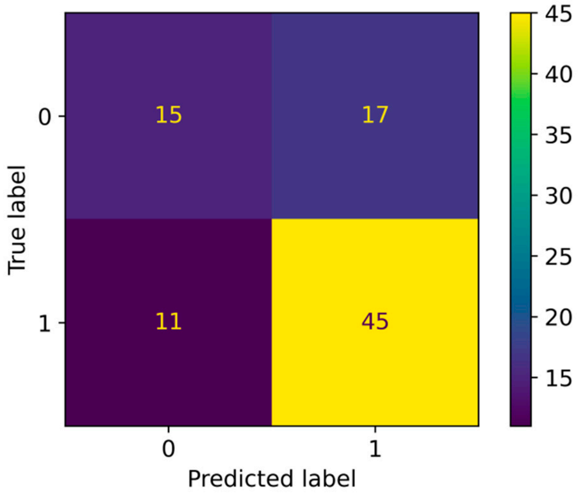

(**a**)

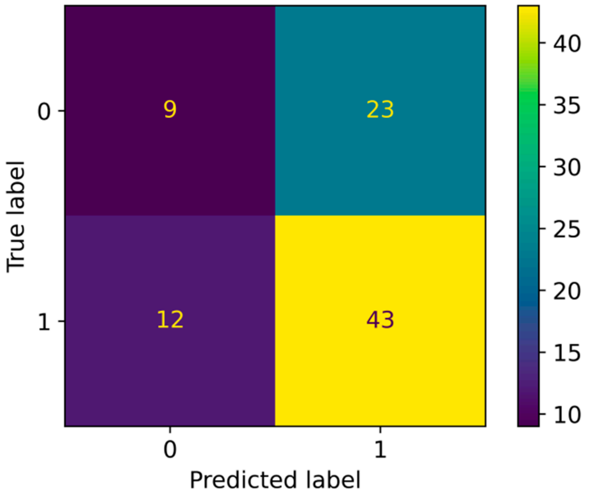

(**b**)

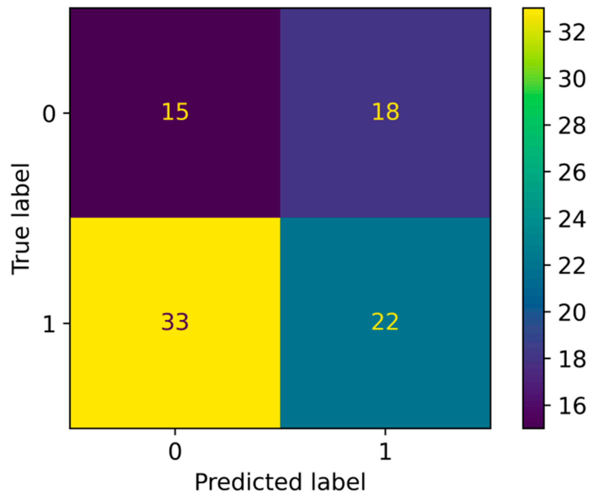

(**c**)

Figure S4. Confusion matrixes calculated from predictions of the tested models on the CBZ - coformer dataset: (a) CCGNet, (b) Method A, (c) HBP.

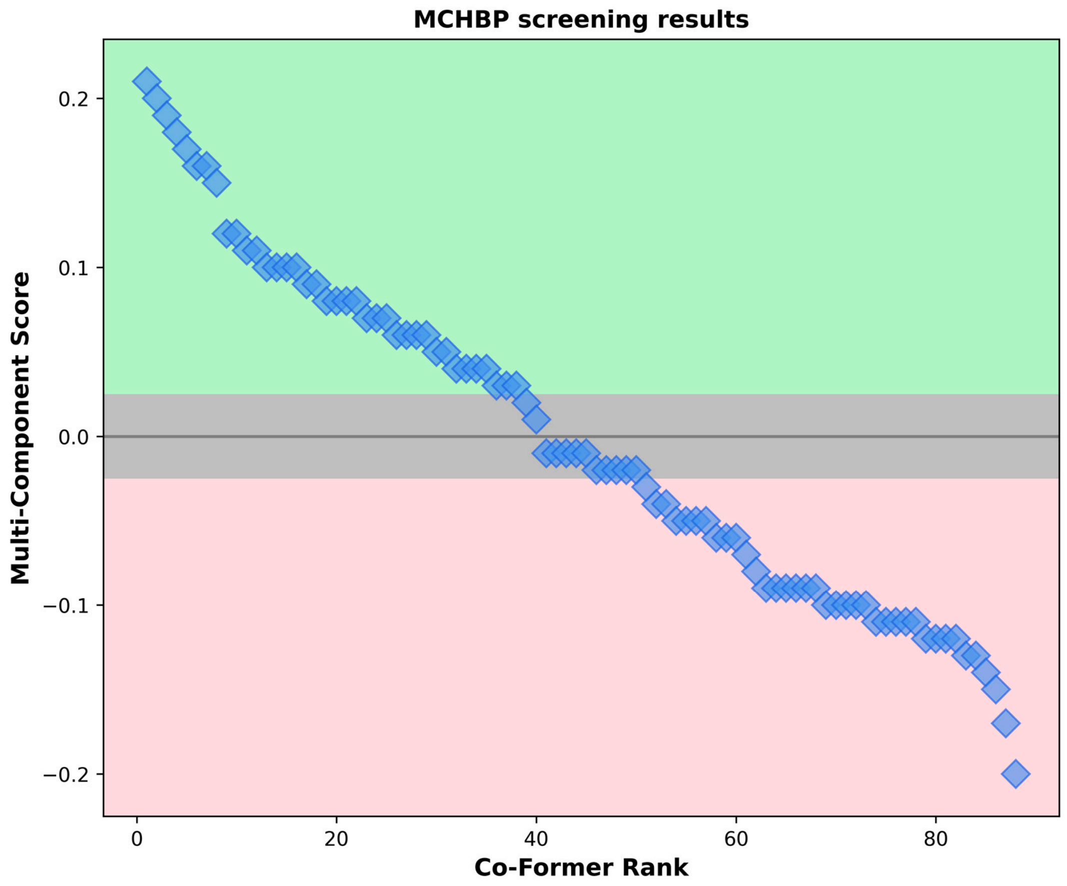

Figure S5. Multicomponent hydrogen-bond propensity chart for CBZ and selected coformers.

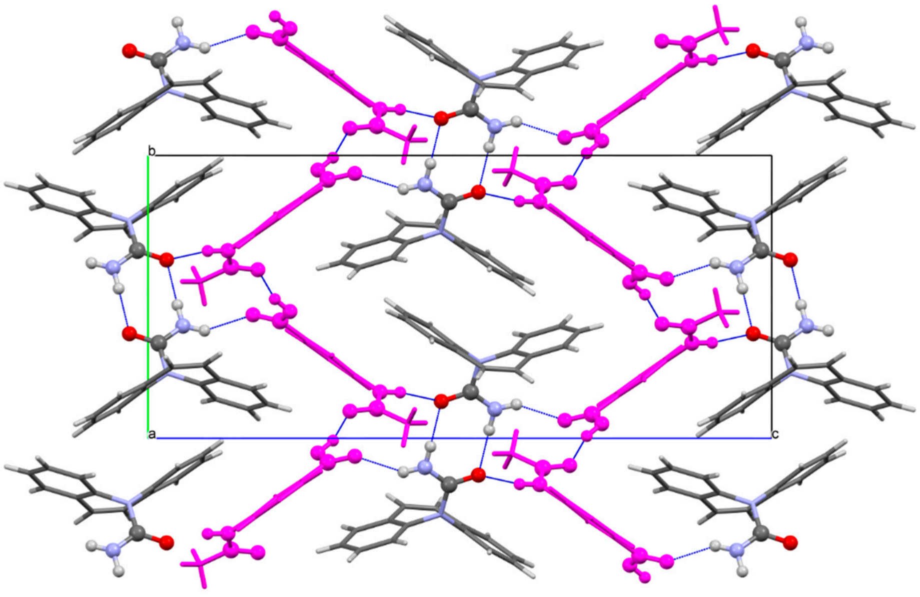

(**a**)

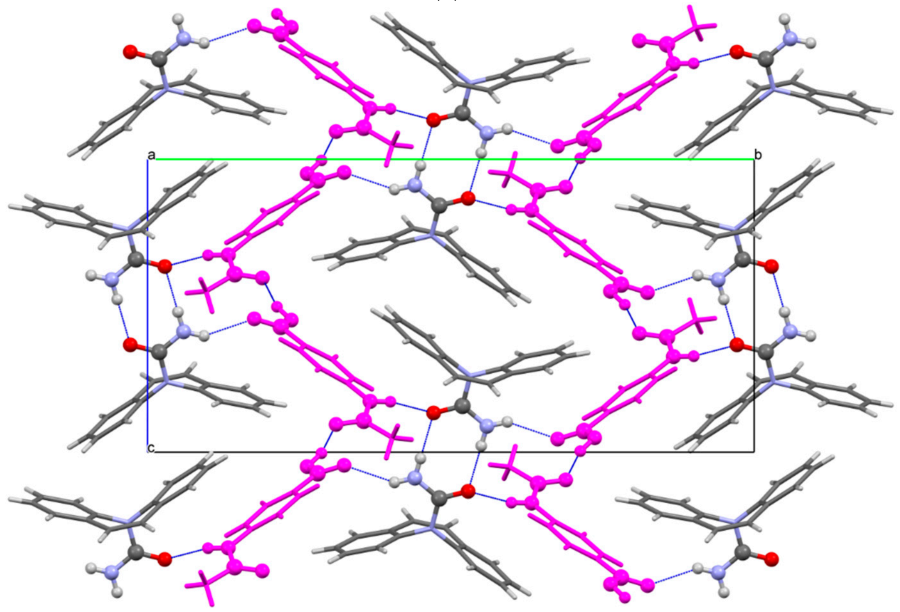

(**b**)

Figure S6. Packing of CBZ and coformer molecules (colored magenta) in the crystal structures of [CBZ+3AcAmBA] (1:1) (a) and [CBZ+4AcAmBA] (1:1) (b).

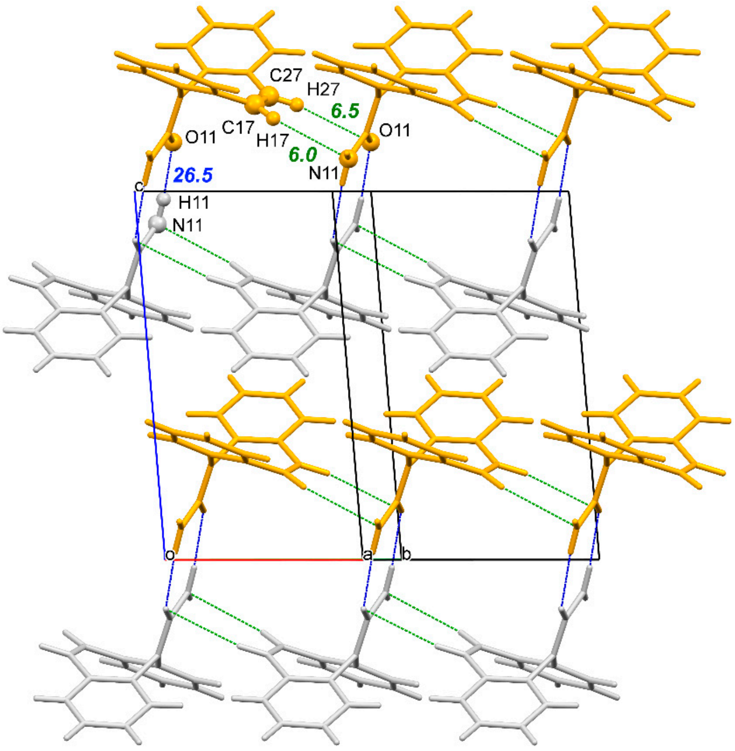

Figure S7. Packing of CBZ dimers in [CBZ+4AcAmBA] (1:1). The molecules are colored by symmetry operation and the numbers indicate the energies of a particular non-covalent interaction estimated according to the QTAIMC scheme in kJ·mol^{-1}.

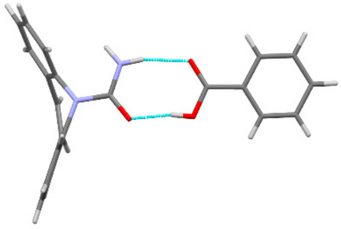

(**a**)

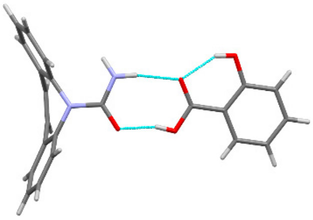

(**b**)

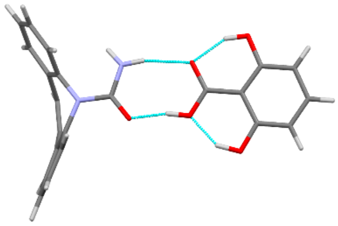

(**c**)

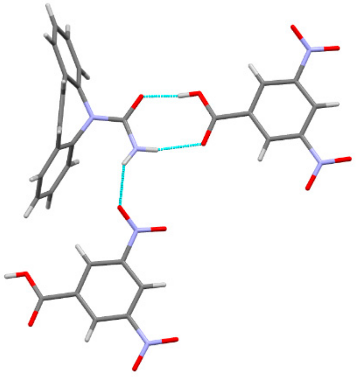

(**d**)

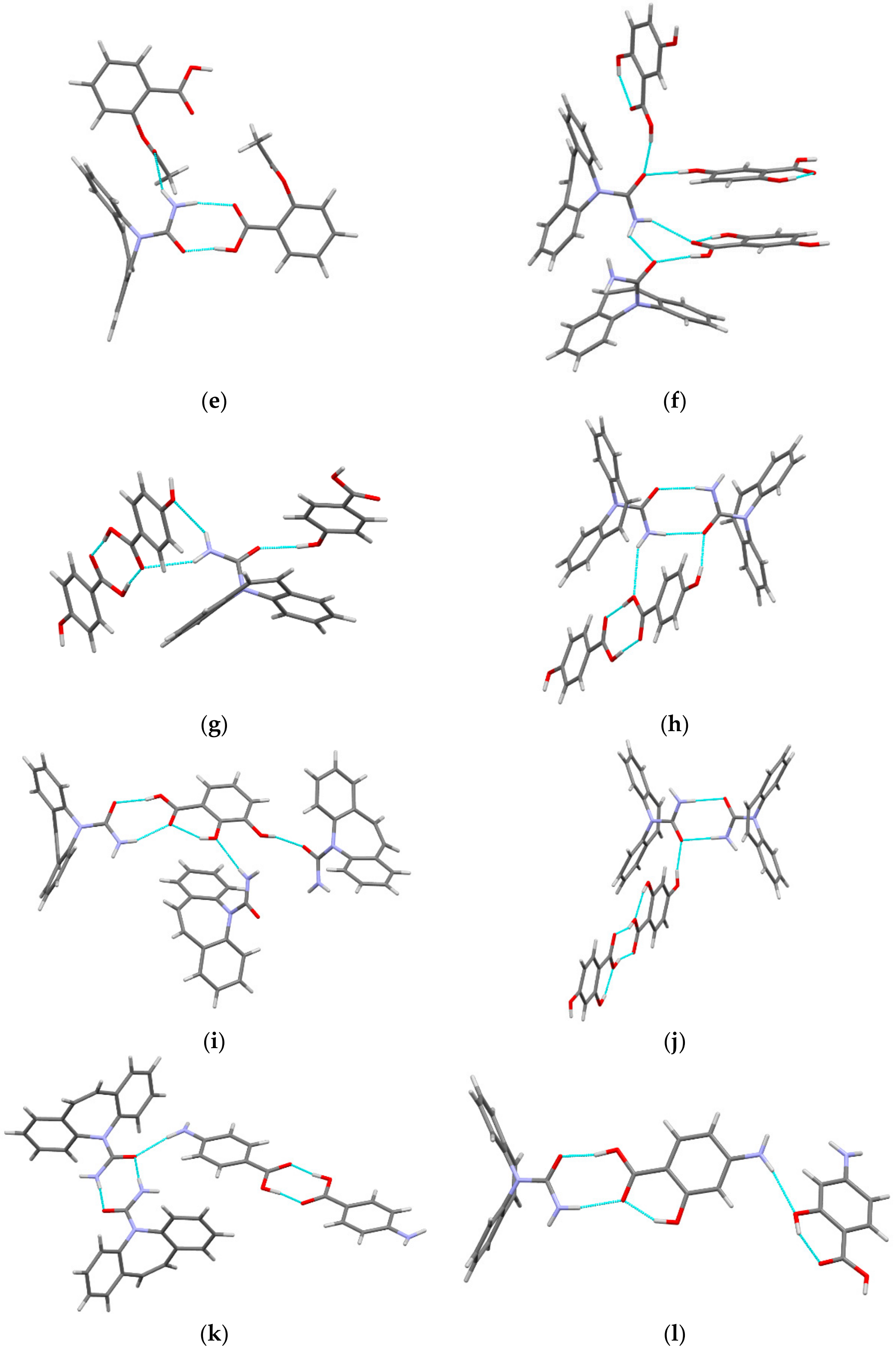

Figure S8. Illustration of hydrogen bonding motifs in the CBZ cocrystals (1:1) with benzoic acid (a), salicylic acid (b), 2,6-dihydroxybenzoic acid (c), 3,5-dinitrobenzoic acid (d), acetylsalicylic acid (e), 2,5-dihydroxybenzoic acid (f), 4-hydroxybenzoic acid (stable form) (g), 4-hydroxybenzoic acid (metastable form) (h), 2,3-dihydroxybenzoic acid (i), 2,4-dihydroxybenzoic acid (j), 4-aminobenzoic acid (k), 4-aminosalicylic acid (l).

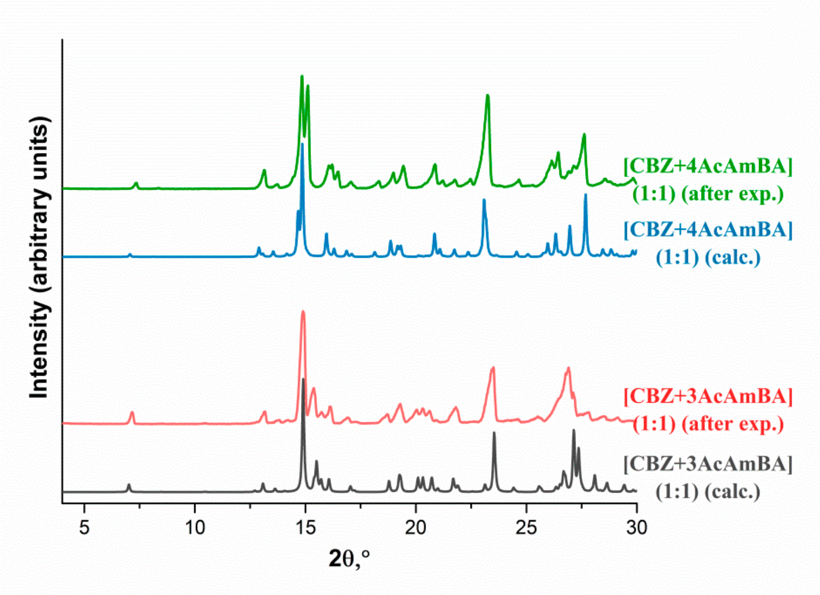

Figure S9. Experimental PXRD patterns of the [CBZ+3AcAmBA] and [CBZ+4AcAmBA] cocrystals and residual materials after long-term equilibrium experiment in organic solvent (methanol or acetonitrile).

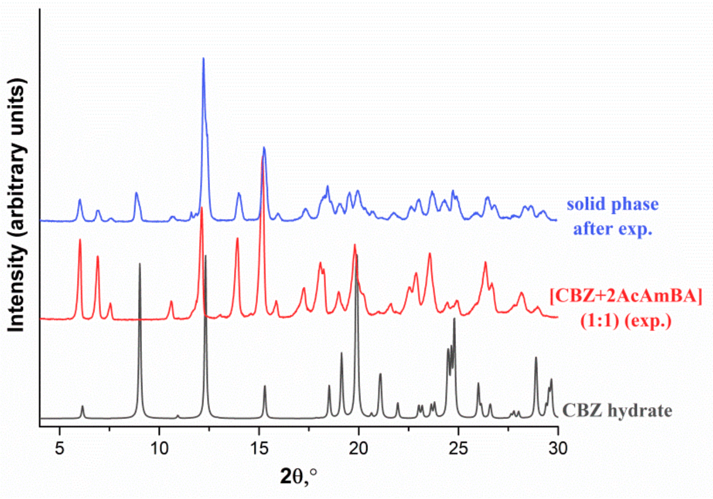

(a)

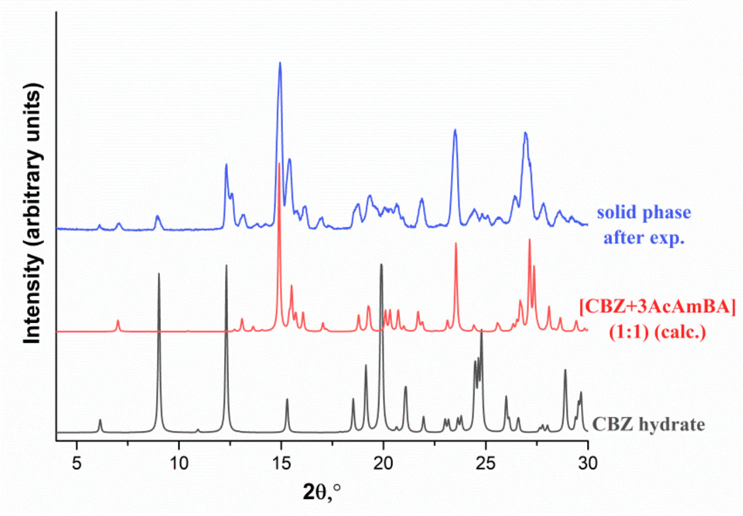

(b)

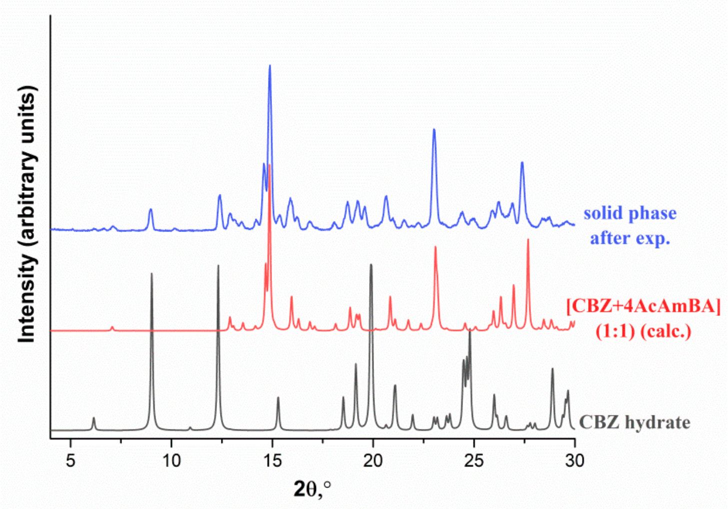

(c)

Figure S10. Experimental PXRD patterns of [CBZ+2AcAmBA] (1:1) (a), [CBZ+3AcAmBA] (1:1) (b), [CBZ+4AcAmBA] (1:1) (c) and solid phases at the eutectic point in a pH 2.0 buffer solution, indicating that both drug and cocrystal solids are presented in equilibrium with the solution.

References:

1. Sathisaran, I. and S.V. Dalvi, *Investigating Cocrystallization of Carbamazepine with Structurally Compatible Coformers: New Cocrystal and Eutectic Phases with Enhanced Dissolution.* AAPS PharmSciTech, 2021. **22**(1): p. 29.
2. Childs, S.L., N. Rodríguez-Hornedo, L.S. Reddy, A. Jayasankar, C. Maheshwari, L. McCausland, R. Shipplett, and B.C. Stahly, *Screening strategies based on solubility and solution composition generate pharmaceutically acceptable cocrystals of carbamazepine.* CrystEngComm, 2008. **10**(7): p. 856-864.
3. Sugden, I.J., D.E. Braun, D.H. Bowskill, C.S. Adjiman, and C.C. Pantelides, *Efficient Screening of Coformers for Active Pharmaceutical Ingredient Cocrystallization.* Crystal Growth & Design, 2022. **22**(7): p. 4513-4527.
4. Sathisaran, I. and S.V. Dalvi, *Cocrystallization of carbamazepine with amides: Cocrystal and eutectic phases with improved dissolution.* Journal of Molecular Structure, 2019. **1193**: p. 398-415.
5. Roca-Paixão, L., N.T. Correia, and F. Affouard, *Affinity prediction computations and mechanosynthesis of carbamazepine based cocrystals.* CrystEngComm, 2019. **21**(45): p. 6991-7001.
6. Huang, D., H.C.S. Chan, Y. Wu, L. Li, L. Zhang, Y. Lv, X. Yang, and Z. Zhou, *Phase solubility investigation and theoretical calculations on drug-drug cocrystals of carbamazepine with Emodin, Paeonol.* Journal of Molecular Liquids, 2021. **329**: p. 115604.
7. Yamamoto, K., S. Tsutsumi, and Y. Ikeda, *Establishment of cocrystal cocktail grinding method for rational screening of pharmaceutical cocrystals.* International Journal of Pharmaceutics, 2012. **437**(1): p. 162-171.
8. Habgood, M., M.A. Deij, J. Mazurek, S.L. Price, and J.H. ter Horst, *Carbamazepine Co-crystallization with Pyridine Carboxamides: Rationalization by Complementary Phase Diagrams and Crystal Energy Landscapes.* Crystal Growth & Design, 2010. **10**(2): p. 903-912.
9. Shayanfar, A., K. Asadpour-Zeynali, and A. Jouyban, *Solubility and dissolution rate of a carbamazepine–cinnamic acid cocrystal.* Journal of Molecular Liquids, 2013. **187**: p. 171-176.
10. Good, D.J. and N. Rodríguez-Hornedo, *Solubility Advantage of Pharmaceutical Cocrystals.* Crystal Growth & Design, 2009. **9**(5): p. 2252-2264.
11. Surov, A.O., K.A. Solanko, A.D. Bond, A. Bauer-Brandl, and G.L. Perlovich, *Cocrystals of the antiandrogenic drug bicalutamide: screening, crystal structures, formation thermodynamics and lattice energies.* CrystEngComm, 2016. **18**(25): p. 4818-4829.
12. Surov, A.O., T.V. Volkova, A.V. Churakov, A.N. Proshin, I.V. Terekhova, and G.L. Perlovich, *Cocrystal formation, crystal structure, solubility and permeability studies for novel 1,2,4-thiadiazole derivative as a potent neuroprotector.* European Journal of Pharmaceutical Sciences, 2017. **109**: p. 31-39.
13. Surov, A.O., A.P. Voronin, A.A. Simagina, A.V. Churakov, S.Y. Skachilova, and G.L. Perlovich, *Saccharin salts of biologically active hydrazone derivatives.* New Journal of Chemistry, 2015. **39**(11): p. 8614-8622.
14. Drozd, K.V., A.N. Manin, and G.L. Perlovich, *Comparative analysis of experimental methods for determining thermodynamic parameters of formation of multi-component molecular crystals: Benefits and limitations.* Journal of Molecular Liquids, 2019. **295**: p. 111644.
15. Oliveira, M.A., M.L. Peterson, and R.J. Davey, *Relative Enthalpy of Formation for Co-Crystals of Small Organic Molecules.* Crystal Growth & Design, 2011. **11**(2): p. 449-457.

16. Ma, K., Y. Zhang, H. Kan, L. Cheng, L. Luo, Q. Su, J. Gao, Y. Gao, and J. Zhang, *Thermodynamic and Kinetic Investigation on the Crucial Factors Affecting Adefovir Dipivoxil-Saccharin Cocrystallization.* Pharmaceutical Research, 2014. **31**(7): p. 1766-1778.
17. Évora, A.O.L., C.E.S. Bernardes, M.F.M. Piedade, A.C.L. Conceição, and M.E. Minas da Piedade, *Energetics of Glycine Cocrystal or Salt Formation with Two Regioisomers: Fumaric Acid and Maleic Acid.* Crystal Growth & Design, 2019. **19**(9): p. 5054-5064.
18. Liu, C., C. Wang, S. Wan, L. Liu, C.C. Sun, and F. Qian, *An Elusive Drug–Drug Cocrystal Prepared Using a Heteroseeding Strategy.* Crystal Growth & Design, 2021. **21**(10): p. 5659-5668.
19. Surov, A.O., A.P. Voronin, K.V. Drozd, T.V. Volkova, N. Vasilev, D. Batov, A.V. Churakov, and G.L. Perlovich, *Extending the Range of Nitrofurantoin Solid Forms: Effect of Molecular and Crystal Structure on Formation Thermodynamics and Physicochemical Properties.* Crystal Growth & Design, 2022. **22**(4): p. 2569-2586.
20. Zhang, S.-W., A.P.J. Brunskill, E. Schwartz, and S. Sun, *Celecoxib–Nicotinamide Cocrystal Revisited: Can Entropy Control Cocrystal Formation?* Crystal Growth & Design, 2017. **17**(5): p. 2836-2843.
21. Ahuja, D., M. Svärd, and Å.C. Rasmuson, *Investigation of solid–liquid phase diagrams of the sulfamethazine–salicylic acid co-crystal.* CrystEngComm, 2019. **21**(18): p. 2863-2874.
22. Manin, A.N., D.E. Boycov, O.R. Simonova, T.V. Volkova, A.V. Churakov, and G.L. Perlovich, *Formation Thermodynamics of Carbamazepine with Benzamide, Para-Hydroxybenzamide and Isonicotinamide Cocrystals: Experimental and Theoretical Study.* Pharmaceutics, 2022. **14**(9): p. 1881.
